# Advisory Committee on Immunization Practices Recommended Immunization Schedule for Children and Adolescents Aged 18 Years or Younger — United States, 2024

**DOI:** 10.15585/mmwr.mm7301a2

**Published:** 2024-01-11

**Authors:** A. Patricia Wodi, Neil Murthy, Veronica V. McNally, Matthew F. Daley, Sybil Cineas

**Affiliations:** ^1^Immunization Services Division, National Center for Immunization and Respiratory Diseases, CDC; ^2^Franny Strong Foundation, West Bloomfield, Michigan; ^3^Institute for Health Research, Kaiser Permanente Colorado, Aurora, Colorado; ^4^The Warren Alpert Medical School of Brown University, Providence, Rhode Island.

At its October 2023 meeting, the Advisory Committee on Immunization Practices* (ACIP) approved the Recommended Child and Adolescent Immunization Schedule for Ages 18 Years or Younger, United States, 2024. The child and adolescent immunization schedule, which can be found on the CDC immunization schedule website (https://www.cdc.gov/vaccines/schedules), is published annually to consolidate and summarize updates to ACIP recommendations on the vaccination of children and adolescents and to assist health care providers in implementing current ACIP recommendations. The 2024 immunization schedule includes several changes to the cover page, tables, notes, and appendix from the 2023 immunization schedule.^†^ In addition, the 2024 child and adolescent immunization schedule includes a new addendum section to summarize new or updated ACIP recommendations that will occur before the next annual update to the child and adolescent immunization schedule. Health care providers are advised to use the cover page, tables, notes, appendix, and addendum together to identify the recommended immunizations for patient populations.

The 2024 child and adolescent immunization schedule is recommended by ACIP (https://www.cdc.gov/vaccines/acip) and approved by CDC (https://www.cdc.gov), the American Academy of Pediatrics (https://www.aap.org), the American Academy of Family Physicians (https://www.aafp.org/home.html), the American College of Obstetricians and Gynecologists (https://www.acog.org/), the American College of Nurse-Midwives (https://www.midwife.org), the American Academy of Physician Associates (https://www.aapa.org), and the National Association of Pediatric Nurse Practitioners (https://www.napnap.org).

ACIP’s recommendations for the use of each vaccine and other immunizing agents are developed after in-depth reviews of product-related data, including the epidemiology and societal impacts of the vaccine-preventable disease, efficacy and effectiveness of the vaccine or other immunizing agent, safety of the vaccine or other immunizing agent, quality of evidence, feasibility of program implementation, impact on health equity, and economic analyses of immunization policy (*1*,*2*). Health care providers should be aware that changes in recommendations for specific vaccines and related agents occur between these annual updates to the child and adolescent immunization schedule.^§^ Such changes will be summarized in the new addendum section; however, health care providers are encouraged to refer to ACIP vaccine recommendations for detailed guidance on the use of each product (https://www.cdc.gov/vaccines/hcp/acip-recs). An online version of the 2024 child and adolescent immunization schedule and instructions for downloading the schedule app are available on the immunization schedule website (https://www.cdc.gov/vaccines/schedules). The use of trade names in the child and adolescent immunization schedule and in this report is for identification purposes only and does not imply endorsement by ACIP or CDC.

## Changes in the 2024 Child and Adolescent Immunization Schedule

Changes to the recommendations for vaccines and related agents in the 2024 immunization schedule for children and adolescents aged ≤18 years include new or updated recommendations for influenza vaccine (*3*), pneumococcal vaccines (*4*), respiratory syncytial virus monoclonal antibody (RSV-mAb) (*5*), respiratory syncytial virus vaccines (RSV) (*6*), COVID-19 vaccines (*7*), inactivated poliovirus vaccine (IPV) (*8*), Mpox vaccine (Mpox) (https://www.cdc.gov/vaccines/acip/meetings/downloads/slides-2023-10-25-26/04-MPOX-Rao-508.pdf), and meningococcal serogroups A, B, C, W, Y vaccine (MenACWY-TT/MenB-FHbp) (https://www.cdc.gov/vaccines/acip/recommendations.html). Diphtheria and tetanus toxoid adsorbed vaccine (DT), 13-valent pneumococcal conjugate vaccine (PCV13), bivalent COVID-19 mRNA vaccines, and meningococcal serogroups A, C, W, Y polysaccharide diphtheria toxoid conjugate vaccine (MenACWY-D, Menactra) were deleted from all sections of the schedule, because these products are no longer distributed or recommended for use in children and adolescents in the United States.

Other changes include clarification of the recommendations for diphtheria, tetanus, and acellular pertussis vaccine (DTaP), *Haemophilus influenzae* type b vaccine (Hib), human papillomavirus vaccine (HPV), measles, mumps, and rubella vaccine (MMR), serogroup B meningococcal vaccine (MenB), and tetanus, diphtheria, and acellular pertussis vaccine (Tdap). Substantial revisions were made to Table 3, which outlines the immunization schedule by medical indication. The definitions for the legend colors were revised to better highlight additional vaccination recommendations for each medical condition and to harmonize with the adult immunization schedule. Finally, a new addendum section was added, which will list new and updated ACIP recommendations that occur before the next annual update to the child and adolescent immunization schedule.

### Cover page

• In the table of abbreviations and trade names, the column header was changed from “vaccine” to “vaccines and other immunizing agents” to account for the inclusion of the newly licensed RSV monoclonal antibody (nirsevimab).

• A sixth step in the “How to Use the Child and Adolescent Immunization Schedule” box was added directing health care providers to review the new Addendum section that lists new or updated ACIP recommendations that occur before the next annual update of the child and adolescent immunization schedule.

• 20-valent pneumococcal conjugate vaccine (PCV20), RSV-mAb (nirsevimab), RSV for maternal vaccination (Abrysvo), Mpox (Jynneos), and pentavalent meningococcal vaccine (MenACWY-TT/MenB-FHbp, [Penbraya]) have been added to the table listing abbreviations and trade names of vaccines and other immunizing agents.

• Diphtheria and Tetanus Toxoid Adsorbed vaccine (DT), 13-valent pneumococcal conjugate vaccine (PCV13), MenACWY-D (Menactra), and bivalent mRNA COVID-19 vaccines were removed from the table listing abbreviations and trade names of vaccines and other immunizing agents, because they are no longer distributed or recommended for use in the United States.

### Table 1 (Routine Immunization Schedule)

• The column header was changed from “vaccine” to “vaccines and other immunizing agents” to account for the inclusion of the newly licensed RSV monoclonal antibody (nirsevimab).

• **COVID-19 row:** The text overlay was revised to reflect updated vaccination recommendations. This text overlay now states, “1 or more doses of updated (2023–2024 Formula) vaccine.”

• **MenACWY row:** Menactra has been deleted.

• **Mpox row**: A new row was added for Jynneos with the column for age 18 years highlighted in purple reflecting the risk-based recommendation for this age group.

• **Pneumococcal conjugate row:** PCV20 has been added and PCV13 has been deleted.

• **Pneumococcal polysaccharide vaccine (PPSV23) row:** This row has been deleted because PPSV23 is no longer routinely recommended for all children and adolescents aged ≥2 years at increased risk for invasive pneumococcal disease. It is still recommended in certain circumstances.

• **RSV-mAb row:** A new row has been added with the columns for ages birth–7 months highlighted in yellow to indicate the recommended age for routine immunization. The overlaying text, “1 dose depending on maternal RSV vaccination status” was also added. In addition, age 8–19 months is highlighted in purple to reflect the risk-based recommendation for this age group.

• **RSV row:** A new row was added for Abrysvo (Pfizer Inc.) and ages 11–18 years are highlighted in purple with the overlaying text, “Seasonal administration during pregnancy” added to reflect the recommendation for the use of Abrysvo (Pfizer Inc.) during pregnancy.

### Table 2 (Catch-up Immunization Schedule)

• **DTaP row:** Language for the minimum interval between doses 4 and 5 was added to clarify when a fifth dose is indicated. The text reads, “A fifth dose is not necessary if the fourth dose was administered at age ≥4 years and ≥6 months after dose 3.”

• **MenACWY row:** Menactra has been deleted.

### Table 3 (Immunization by Medical Indication Schedule)

• A sentence was added to the header of Table 3 stating that medical conditions are often not mutually exclusive and that health care providers should review all relevant columns in the Table if multiple conditions are present.

• The column header was changed from “vaccine” to “vaccines and other immunizing agents” to account for the inclusion of the newly licensed RSV monoclonal antibody (nirsevimab).

• **Legend:** The definitions of the yellow, purple, and gray colors boxes in the legend were revised. Based on the revised definitions, the colors for many of the rows in this table have changed. In addition, the checked yellow color was changed to a brown color to harmonize with the 2024 adult immunization schedule.

• **Mpox row:** A new row was added for Jynneos. Across all medical indications listed, the entire row is purple reflecting the risk-based recommendation for Mpox vaccination. In the pregnancy column, an overlaying text, “See Notes” has been added, directing health care providers to review the pregnancy bullet in the Mpox vaccination notes.

• **RSV-mAb row:** A new row was added to summarize nirsevimab immunization recommendations by medical condition. The columns for both immunocompromised status (excluding HIV infection) and HIV infection with CD4 <15% or <200 cells per mm^3^ is highlighted in brown and an overlaying text “2nd RSV season” was added. In addition, the column for heart disease or chronic lung disease is also highlighted in brown with the overlaying text “2nd RSV season for chronic lung disease.”

• **RSV row:** A new row was added for use of Abrysvo (Pfizer Inc.) during 32–36 weeks’ gestation. The pregnancy column is highlighted in yellow with overlaying text of “seasonal administration” added to indicate that the maternal RSV vaccination recommendation is on the basis of RSV seasonality.

### Vaccine Notes

The notes for each vaccine and related agent are presented in alphabetical order. Edits have been made throughout the Notes section to harmonize language, to the greatest extent possible, with that in the adult immunization schedule.

• **Additional information:** The text for vaccine injury compensation was revised to add Mpox and RSV to the list of vaccines not covered by the National Vaccine Injury Compensation Program. Mpox is covered by the Countermeasures Injury Compensation Program.

• **COVID-19:** The language in the “Routine vaccination” and “Special situations” sections was revised to reflect the current COVID-19 vaccination recommendations for children and adolescents. The number of doses needed and intervals between doses might vary on the basis of a patient’s previous vaccination history, immunocompromised status, and the vaccine product used. The “Routine vaccination” section describes the recommendations for the general population, and the “Special situations” section describes the recommendations for persons who are moderately or severely immunocompromised. In addition, hyperlinks to the current COVID-19 vaccination schedules as well as Emergency Use Authorization indications for COVID-19 vaccines are included.

• **DTaP:** Language in the “Routine vaccination” section was revised to clarify primary and booster doses.

• **HPV:** In the “Routine vaccination” section, the recommendation for interrupted schedules was removed because that information is also presented on the Cover Page and applicable to all vaccines. In addition, to improve clarity, the words, “of any valency” were added to the bullet, “No additional dose recommended when any HPV vaccine series *of any valency* has been completed using the recommended dosing intervals.”

• **Influenza:** A hyperlink to the 2023–24 influenza recommendations and a bullet for the 2024–25 influenza recommendations were added. In the “Special situations” section, all bullets describing recommendations for persons with a history of egg allergy were removed. Persons with a history of egg allergy of any severity can be vaccinated with any influenza vaccine indicated for the recipient’s age and health status, with no additional safety considerations. A note describing this recommendation was added at the end of the “Special situations” section.

• **MMR:** The bullet, “If MMRV is used, the minimum interval between MMRV doses is 3 months” was moved to the end of the notes section. In addition, the “Routine vaccination,” “Catch-up vaccination,” and “Special situations” sections were revised to clarify that this minimal interval is applicable to all sections.

• **MenACWY**: All reference to Menactra was removed because this vaccine is no longer distributed in the United States, and any remaining doses of this product expired in October 2023. In addition, information about the use of the newly licensed pentavalent meningococcal vaccine (Penbraya) is included at the end of the MenACWY notes.

• **MenB**: A note summarizing recommendations for Penbraya was added. In addition, a link to a resource to assist health care providers with shared clinical decision-making recommendations for MenB vaccination was added.

• **Mpox:** A new section describing the recommendations for use of Jynneos in adolescents aged 18 years, including sexual risk factors and vaccination during pregnancy, was added.

• **Pneumococcal:** The “Routine vaccination,” “Catch-up vaccination,” and “Special situations” sections have been updated with the new recommendations for use of 15-valent pneumococcal conjugate vaccine (PCV15), PCV20, and PPSV23. PCV13 was deleted from all sections. Chronic kidney disease, chronic liver disease, and moderate persistent or severe persistent asthma were added to the list of medical conditions that increase the risk for invasive pneumococcal disease.

• **Poliovirus:** The “Catch up vaccination” section has been revised to include updated recommendations for adolescents aged 18 years. Language was added stating that most adolescents aged 18 years who were born and raised in the United States can assume to be vaccinated against poliovirus as children. The “Special situations” section was revised to describe administering a one-time, lifetime IPV booster to adolescents aged 18 years who have completed the primary series and are at increased risk for exposure to poliovirus.

• **RSV-mAb**: A new section was added to provide details on the use of nirsevimab in infants and young children. The “Routine immunization” section outlines the recommendations for infants aged <8 months. The “Special situations” section describes recommendations for age-eligible children who are undergoing cardiac surgery with cardiopulmonary bypass, and children aged 8–19 months who are at increased risk for severe RSV disease. Information describing timing of immunization, including guidance for jurisdictions with RSV seasonality that differs from most of the continental United States, was included.

• **RSV**: A new section was added outlining recommendations for maternal RSV vaccination with Abrysvo (Pfizer Inc.) using seasonal administration. Language was added to clarify that health care providers should take one of two approaches to prevent severe respiratory syncytial virus disease in infants: either administer Abrysvo (Pfizer Inc.) to pregnant persons at 32–36 weeks’ gestation or administer nirsevimab to the infant. Information describing vaccination timing, including guidance for jurisdictions with RSV seasonality that differs from most of the continental United States, was included.

• **Tdap**: The “Routine vaccination” and “Catch-up vaccination” sections were revised to clarify that the Tdap dose recommended at age 11–12 years is the adolescent Tdap booster dose.

### Appendix (Contraindications and Precautions)

• The header sentence of the Appendix was revised to include all the sources used to create the Appendix.

• The column header was changed from “Vaccine” to “Vaccines and other immunizing agents” to account for the inclusion of the newly licensed RSV monoclonal antibody (nirsevimab).

• **COVID-19 row**: Two new rows for COVID-19 vaccines were added to describe contraindications and precautions to COVID-19 vaccination. The first row lists contraindications and precautions to receipt of mRNA vaccines (Pfizer-BioNTech and Moderna), and the second row lists contraindications and precautions to receipt of the protein subunit vaccine (Novavax).

• **DTaP and DT row**: DT was deleted because this vaccine is no longer distributed in the United States.

• **Hib row**: In the “Contraindicated or Not Recommended” column, the bullet describing history of severe allergic reaction to dry natural latex was removed because most vials of Hib products no longer contain latex.

• **Meningococcal ACWY row**: Menactra was removed because this product is no longer distributed in the United States. Any remaining doses expired in October 2023.

• **Meningococcal ABCWY row**: A new row was added to describe contraindications and precautions to vaccination with the new pentavalent meningococcal vaccine, Penbraya.

• **RSV-mAb row**: A new row for nirsevimab was added to describe contraindications and precautions to nirsevimab.

• **RSV row**: A new row for RSV (Abrysvo [Pfizer Inc.]) was added describing the contraindications and precautions to RSV vaccination.

### Addendum

A new Addendum section was added to the child and adolescent immunization schedule to summarize new and updated ACIP recommendation(s) that occur before the next annual update to the child and adolescent immunization schedule.

### Additional Information

The Recommended Child and Adolescent Immunization Schedule, United States, 2024 is available at https://www.cdc.gov/vaccines/schedules/hcp/imz/child-adolescent.html. The full ACIP recommendations for each vaccine are also available at https://www.cdc.gov/vaccines/hcp/acip-recs. All vaccines and immunizing agents identified in Tables 1, 2, and 3 (except DTaP, rotavirus, and nirsevimab) also appear in the Recommended Adult Immunization Schedule for Ages 19 Years or Older, United States, 2024, available at https://www.cdc.gov/vaccines/schedules/hcp/imz/adult.html. The notes and appendix for vaccines that appear in both the child and adolescent immunization schedule and the adult immunization schedule have been harmonized to the greatest extent possible.

## Footnotes

* Recommendations for routine immunization of children and adolescents are developed by ACIP, a federal advisory committee chartered to provide expert external advice and guidance to the CDC director on use of vaccines and related agents for the control of vaccine-preventable diseases in the civilian population of the United States. Recommendations for routine immunization of children and adolescents are harmonized to the greatest extent possible with recommendations made by the American Academy of Pediatrics, the American Academy of Family Physicians, the American College of Obstetricians and Gynecologists, the American College of Nurse-Midwives, the American Academy of Physician Associates, and the National Association of Pediatric Nurse Practitioners. ACIP recommendations become official agency guidelines once the recommendation has been adopted by the CDC Director. Additional information about ACIP is available at https://www.cdc.gov/vaccines/acip.

† Past immunization schedules are available at https://www.cdc.gov/vaccines/schedules/hcp/schedule-related-resources.html.

§ CDC encourages organizations to use syndication as a more reliable method for displaying the most current and accurate immunization schedules on an organization’s website rather than copying these schedules to their websites. Use of content syndication requires a one-time step that ensures an organization’s website displays current schedules as soon as they are published or revised; instructions for syndication code are available on CDC’s website (https://www.cdc.gov/vaccines/schedules/resource-library/syndicate.html). CDC also offers technical assistance for implementing this form of content syndication (requests can be emailed to ncirdwebteam@cdc.gov).
